# ACDC, a global database of amphibian cytochrome-b sequences using reproducible curation for GenBank records

**DOI:** 10.1038/s41597-020-00598-9

**Published:** 2020-08-13

**Authors:** Matthijs P. van den Burg, Salvador Herrando-Pérez, David R. Vieites

**Affiliations:** 1 Department of Biogeography and Global Change. Museo Nacional de Ciencias Naturales (MNCN), Consejo Superior de Investigaciones Científicas (CSIC), C/José Gutiérrez Abascal 2, 28006 Madrid, Spain; 2 Institute of Biodiversity and Ecosystem Dynamics (IBED), University of Amsterdam, Amsterdam, The Netherlands; 3 School of Biological Sciences, The University of Adelaide, 5005 South Australia, Australia

**Keywords:** Taxonomy, Evolutionary biology, Genetic markers, Phylogenetics

## Abstract

Genetic data are a crucial and exponentially growing resource across all biological sciences, yet curated databases are scarce. The widespread occurrence of sequence and (meta)data errors in public repositories calls for comprehensive improvements of curation protocols leading to robust research and downstream analyses. We collated and curated all available GenBank cytochrome-b sequences for amphibians, a benchmark marker in this globally declining vertebrate clade. The Amphibia’s Curated Database of Cytochrome-b (ACDC) consists of 36,514 sequences representing 2,309 species from 398 genera (median = 2 with 50% interquartile ranges of 1–7 species/genus). We updated the taxonomic identity of >4,800 sequences (ca. 13%) and found 2,359 (6%) conflicting sequences with 84% of the errors originating from taxonomic misidentifications. The database (accessible at 10.6084/m9.figshare.9944759) also includes an *R* script to replicate our study for other loci and taxonomic groups. We provide recommendations to improve genetic-data quality in public repositories and flag species for which there is a need for taxonomic refinement in the face of increased rate of amphibian extinctions in the Anthropocene.

## Abstract (summary)

**Table:** 

Measurement(s)	DNA • mitochondrial_DNA • cytochrome b
Technology Type(s)	digital curation • bioinformatics analysis
Sample Characteristic - Organism	Amphibian

Machine-accessible metadata file describing the reported data: 10.6084/m9.figshare.12587744

## Background & Summary

Genetic data repositories are a key research component across scientific disciplines that rely on genetic sequences correctly assigned to a reference taxonomy. Although mistaken identity and composition of sequences within those repositories have long been acknowledged^1–5^, broad-scale data-quality evaluations remain scarce^6–8^ and rarely translate into improved databases. Therefore, the uncertainty of genetic data in global platforms such as GenBank^3,9,10^ represents a paramount obstacle for robust downstream analyses. Critically, quality-screening efforts can resolve misidentification of known, cryptic and undescribed taxa^8,11^, and inform the definition of reliable taxonomical units for management and biodiversity research^12,13^.

The widespread sequencing of, and access to, mitochondrial DNA (mtDNA) has boosted taxonomic studies via integrative taxonomy, barcoding, bioprospection, phylogenetics, phylogeography, population and conservation genetics, biogeography, macroecology, and paleoecology^14–16^. Available mtDNA data outcompetes nuclear DNA data in taxonomic coverage across the ‘Tree of Life’ mainly due to the popularity of 16 S, cytochrome-b (Cytb) and cytochrome oxidase 1 (Cox1) loci, while multiple sequences per species of those loci have proved crucial to define species limits^17–19^. While Cox1 was proposed as a universal barcode genetic marker^20^, GenBank’s Cytb records are currently more abundant than Cox1 for all five major vertebrate groups (Table 1).

**Table 1:** GenBank records for Cytochrome-b (Cytb) and Cytochrome oxidase subunit I (Cox1) for the main five vertebrate groups.

Organism	Cytb records	Cox1 records
Amphibia	46,116	23,675
Aves	52,136	36,573
Fish	507,149	364,802
Mammalia (except humans)	140,367	58,249
Reptilia	57,998	15,471
**Total**	**803,766**	**498,770**

Search queries (27/07/2019): “cytochrome b OR cytb AND *Class*[Organism]”, and “cytochrome oxidase subunit I OR cox1 AND *Class*[Organism]”. Fish represent Actinopterygii, Sarcopterygii, and Chondrichthyes.

Amphibians have the highest rate of newly discovered vertebrate species^21^ given intense taxonomic efforts^11^. These ectotherms are however the most threatened vertebrates on Earth^22,23^, with many species facing extinction owing to emerging and spreading diseases^24,25^, habitat loss^26^ and climate change^27^. Therefore, accurate phylogenetic identification^11,28,29^ remains critical for future research and conservation actions. Here, we present the Amphibia’s Curated Database of Cytochrome-b sequences (ACDC^30^, 10.6084/m9.figshare.9944759), a comprehensive and curated database of all amphibian Cytb sequences available in GenBank. We targeted Cytb because it is the most common genetic marker, with the broadest genus- and species-level taxonomic coverage, in the amphibian literature^31,32^.

**Fig. 1:**
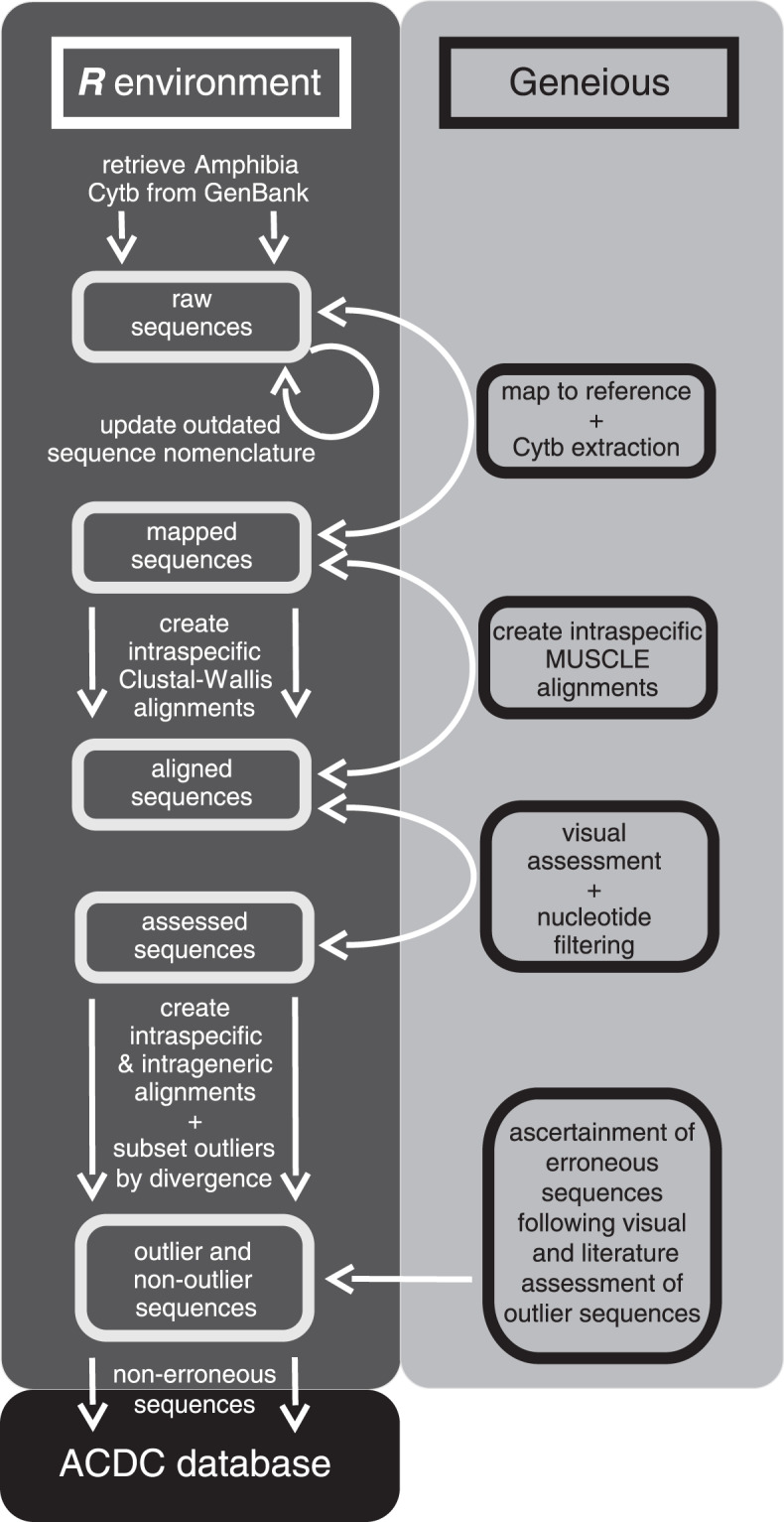
Workflow to collate and curate The Amphibia’s Curated Database of Cytochrome-b sequences (ACDC).

We created ACDC^30^ following a multi-step process implemented in a bioinformatic pipeline combining data retrieval from GenBank, local sequence alignments and quantification of genetic divergences (Fig. 1). On 01 February 2018, we retrieved a total of 39,202 Cytb sequences. Following curation (see Methods), ACDC contains 36,514 unique sequences representing 398 genera and 2,309 species (median = 2 species/genus with 50% interquartile ranges of [1,7]). For 1,363 species and 74 of the 75 amphibian families, there is more than one sequence available (Summary_statistics_ACDC.xlsx^30^) (median = 7 [3,22] species/family). ACDC represents 29% of the 7,963 currently known amphibian species covering most clades^33^. Despite the taxonomic accuracy of GenBank records seems to be accurate above the genus level^34^, our work demonstrates that the problematic issues mostly occur at the species level, and case-by-case assessments of taxonomic identity are necessary.

**Fig. 2:**
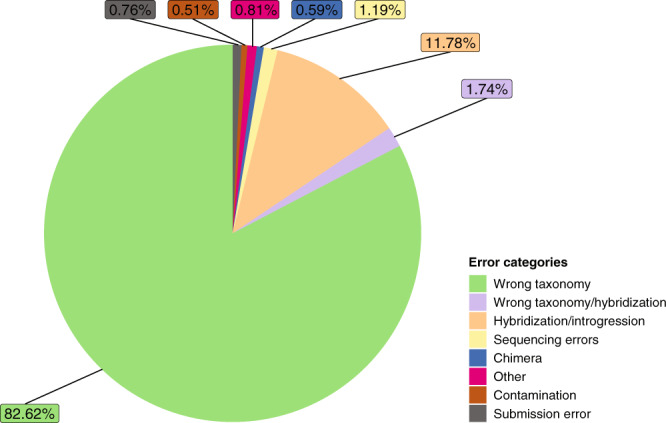
Frequency of error categories in amphibian Cytochrome-b sequences identified from GenBank sequences (01/02/2018). Those errors affect 6% (n = 2,359) of the sequences retrieved. Sequences identified due to incomplete lineage sorting are lumped in ‘Hybridization/Introgression’. Category definitions are explained in Erroneous_sequences.xlsx^30^.

We identified 2,359 conflictive sequences (6% of the collated dataset) from 1,603 Anura, 743 Caudata, and 13 Gymnophiona records. These sequences suffered from wrong taxonomic assignments (>80%), contamination, introgression/hybridization, and submission/ sequencing errors (Fig. 2, Erroneous_sequences.xlsx^30^) and, as such, they qualify to be tagged as ‘UNVERIFIED’^35^ in GenBank. We updated the taxonomic identity of ca. 4,800 GenBank records (Taxonomic_corrections.xlsx^30^), and reverse-complemented reads from >1,000 sequences incorrectly uploaded as backward reads. We provide summary tables listing species/sequences with an uncertain taxonomic assignment (sp./ssp./cf./aff.; Uncertain_taxonomy_to_be_assessed.xlsx^30^) and potentially belonging to species complexes (Species_notes.xlsx^30^). These results suggest that several amphibian groups are in need of taxonomic revision. Lastly, we address general recommendations to improve data quality in public genetic repositories (Table 2) and append an *R* script^30^ to apply our data-curation protocol to other taxa and loci.

**Table 2:** Recommendations to improve the quality of (meta)data reported in GenBank.

	Recommendation	Audience
1.	Create a GenBank’s default notification system whereby data users can report errors and uncertainties to data owners.	Authors GenBank
2.	Change editing restrictions for GenBank’s ‘DEFINITION’ field allowing authorities to make changes under GenBank personnel’s supervision. GenBank could assign specific taxa to specific experts very much like the assessment of the conservation status of target taxa is assigned to working groups by the International Union for Conservation of Nature.	GenBank
3.	Synchronize GenBank-record identity with manuscript identity, especially cf., aff. and unidentified species (e.g., sp. 1/2/3). GenBank could grant a label of excellence to contribute to data improvement and make it available online for curricular purposes.	Authors GenBank
4.	Before submission to GenBank, users should BLAST their sequences against the GenBank database to detect taxonomic inconsistencies, contamination, and identical sequences already available in GenBank. We recommend that all intrageneric alignments are always visually checked using the range of powerful tools available in commercial and free-source genetic software (e.g., CLC Workbench, Geneious, MEGA).	Authors
5.	GenBank should not remain *blasé* about accumulating uncertainty, and instead be proactive to resolve taxonomic vagueness as shown in our study (i.e., 1,836 amphibian sequences currently reported as cf./aff./sp./ssp.; see Uncertain_taxonomy_to_be_assessed.xlsx^30^). Thus, justification of taxonomic assignments above the species level should be part of the data-submission protocol.	GenBank Authors
6.	While improving GenBank reporting etiquette is crucial, how GenBank information is reported in the literature is equally important. Authors should cite in their publications GenBank accession numbers along with full details of each study specimen and sequence (namely sampling locality, specimen identity, assigned phylogenetic clade/lineage/haplotype, and cross-references to published figures/tables). Reporting this information could be enforced as a compulsory requirement for publication by journals and would facilitate data curation in public repositories.	Authors Journal editors

Ideally, the research community would benefit from future sequencing efforts giving full taxonomic coverage to a selected sample of loci, which could in turn improve our understanding of amphibian biodiversity, evolution, ecology or conservation. mtDNA markers are still the best candidates to implement those efforts, as they are easy to amplify (even in poorly preserved samples), align and curate^36^. Taxonomic coverage of mtDNA can also be widened as a by-product of full-transcriptome and -genome assemblage, including long-read Next Generation Sequencing. In that respect, the development, integration, and expansion of quality-curated databases like ACDC should promote the generation of novel genomic data covering multiple specimens per species across the amphibian tree of life.

## Methods

### Workflow

Within the *R* environment^37^, on 01/02/2018, we used a key-word string to select and download all amphibian Cytb sequences from the GenBank’s website (www.ncbi.nlm.nih.gov/genbank, National Centre for Biotechnology Information) – see Steps 1–3 in the ACDCv1.0.R script^30^. We eliminated duplicates using GenBank labels ‘NC’, adjusted the nomenclature of each sequence to conform a genus_species_accession format (e.g., *Bufo_bufo*_AB123456), and exported all sequences as a single *.fasta file (Step 4^30^). This includes single Cytb sequences, as well as mitochondrial genomes that contain this locus. All these sequences were then mapped against a reference mitochondrial genome (*Xenopus tropicalis*, AY789013), using the ‘high sensitivity’ option in Geneious® v11.0^38^, and we extracted Cytb nucleotidic sequences (Fig. 1). Then, the nomenclature of all unique taxonomic identities was compared, confirmed and, if applicable, updated (Step 5^30^) against the Amphibian Species of the World Database^33^.

We exported all mapped Cytb sequences in a *.fasta file from Geneious to the *R* environment. Therein, we performed ClustalW^39^ multiple sequence alignments for each species separately using the *R* package Bioconductor (Step 6^30^). The resulting intraspecific alignments were imported back to Geneious as *.fasta files for batch-alignment through the MUSCLE algorithm (Fig. 1). The former step was mandatory because batch-MUSCLE alignments of multiple sequences (*muscle* function^40^ in Bioconductor) does not reorder sequences based on genetic similarity (A.T. Kalinka, pers. comm., 06/08/2018). Within Geneious, we visually resolved nucleotide gaps using the Vertebrate Mitochondrial Code^41^, and removed sequence ends with ambiguous nucleotides.

### Taxonomic assessment and curation

We quantified accuracy on the assignation of sequences to species based on the genetic divergence (%) among sequences within species and genera and the identification of divergence outliers. We implemented three steps to detect sequencing and taxonomic errors based on pairwise-sequence alignments within each genus (Step 7; see Technical Validation). We used ‘uncorrected divergence’ as the genetic distance between every pair of sequences, using the *seqinr* package^42^. Firstly, we accepted sequences showing ≤3% divergence within multiple alignments across all sequences of the same species, and subset those with >3% divergence for further examination. Secondly, we also accepted sequences showing >3% divergence within a genus and subset those with ≤3% divergence for further examination. We caution that 3% is a reliable (conservative) divergence threshold for amphibian Cytb^43–46^ but should be re-estimated for other loci and taxonomical groups. Thirdly, for all potentially erroneous sequences, we assessed taxonomic and geographical veracity against (I) the data-source publication cited in GenBank, (II) the most recent papers dealing the taxon involved, (III) AmphibiaWeb (https://amphibiaweb.org) and (IV) the Amphibian Species of the World Database^33^ (Fig. 1). References and rationale used to separate erroneous from non-erroneous sequences are given for each sequence (Erroneous_sequences.xlsx^30^). We removed all erroneous sequences from ACDC and compiled all Amphibia Cytb sequences with uncertain taxonomy (aff./cf./sp./ssp.) (Uncertain_taxonomy_to_be_assessed.xlsx^30^). Lastly, the curation of genetic data is dependent on the number of available sequences per species and the taxonomic coverage per genus. Therefore, we included summary data for the ACDC database (Summary_statistics_ACDC.xlsx^30^) to flag species in need of more data and taxonomic resolution in online genetic repositories.

Lastly, our *R* script includes a routine to assess the Cytb region that maximizes species coverage and number of sequences (Supplementary Files 1 and 2). To do so, we first mapped all ACDC sequences to the Cytb of *X*. *tropicalis* (AY789013) using the ‘highest sensitivity’ option in Geneious, then counted non-missing bases for each position (Step 8^30^).

## Data Records

The curated database, all files as well as the associated *R* script are freely available on *figshare*^30^. The database consists of two compressed batches of *.fasta files of species with (I) 1 sequence (Species_with_One_Sequence.zip) and (II) > 1 sequences (Species_with_Multiple_Sequences.zip).

## Technical Validation

We implemented a three-step sequence of filters to assess Cytb-sequence quality. (I) We retained sequences with complete binominal nomenclature. (II) We mapped all sequences against the *Xenopus tropicalis* mitochondrial genome (AY789013) and reverse-complemented sequences incorrectly submitted in backward-read format (>1,000). (III) We visually scanned sequence alignments for sequencing errors, whereby non-amino acid gaps (≠3) were filled or replaced by ‘N’ in the absence or presence of diversity at the base in question, respectively.

## Supplementary information

Supplementary File 1

Supplementary File 2

## Data Availability

The *R* script used to collate and curate the Amphibia Cytb database is available at *figshare* (ACDCv1.0.R^30^).
